# Comparative Outcomes of Percutaneous K-Wires Versus Plate Fixation in the Treatment of Distal Radius Fractures: A Focus on Stability in Osteoporotic Patients and Both-Bone Distal End Fractures

**DOI:** 10.7759/cureus.72981

**Published:** 2024-11-04

**Authors:** Kapil Shinde, Ketan Kantamaneni, Reshmitha Kantamneni, Emad Ahmed, Muhammad Asad Arif, Sravan Sanka, Salih Seidahmed, Christopher James

**Affiliations:** 1 Trauma and Orthopaedics, East Kent Hospitals University NHS Foundation Trust, Ashford, GBR; 2 Trauma and Orthopaedics, Rangaraya Medical College, Kakinada, IND; 3 Orthopedic Surgery, East Kent Hospitals University NHS Foundation Trust, Ashford, GBR; 4 Orthopaedics and Trauma, East Kent Hospitals University NHS Foundation Trust, Ashford, GBR; 5 Orthopaedics, East Kent Hospitals University NHS Foundation Trust, Ashford, GBR; 6 Trauma and Orthopaedics, William Harvey Hospital, Ashford, GBR

**Keywords:** both bone fractures, distal end radius fracture, k-wire fixation, osteoporotic, plate fixation

## Abstract

Background

Distal radius fractures (DRFs) are commonly treated with percutaneous K-wire or plate fixation. The relative efficacy and stability of these methods, particularly in osteoporotic patients and in cases involving both distal radius and ulna fractures, remain subjects of clinical debate.

Objective

This study aims to compare the outcomes of percutaneous K-wire fixation versus plate fixation in patients with distal end radius fractures, focusing on postoperative stability in osteoporotic conditions and both-bone distal end fractures.

Methods

A total of 50 patients were retrospectively analysed and divided into two groups: 25 underwent percutaneous K-wire fixation, and 25 underwent plate fixation. Outcomes measured included bone healing time, complication rates, functional recovery using the DASH score, and radiological outcomes through standard X-ray imaging.

Results

Preliminary data suggest that plate fixation offers better stability, especially in osteoporotic patients and cases with both-bone involvement. Complications with K-wire fixation were more frequent in these subgroups.

Conclusion

Plate fixation may provide superior stability and functional outcomes in treating DRFs, particularly in complex cases involving osteoporosis or bone injuries. Further research with larger sample sizes and prospective design is recommended.

## Introduction

Distal radius fractures (DRFs) are among the most common injuries encountered in orthopaedic practice [1], particularly among the elderly with osteoporotic bones, where bone quality significantly impacts the choice of fixation method and subsequent outcomes. Two prevalent methods for treating DRFs are percutaneous Kirschner wires (K-wires) and plate fixation, each with distinct advantages and limitations regarding stability, functional outcomes, and complication rates [2,3].

Traditional fixation techniques, such as percutaneous K-wire fixation, have been challenged by more modern approaches like plate fixation. These may offer improved stability and outcomes, especially in more complex fracture patterns or compromised bone quality [4]. 

Percutaneous K-wire fixation is often favoured for its minimally invasive nature and relatively straightforward application. This technique involves the insertion of wires through the skin to stabilise the fracture without extensive dissection, which can be particularly advantageous in patients with poor bone quality [5]. K-wire fixation is associated with reduced surgical trauma and shorter operative times but may face challenges in maintaining long-term stability, particularly in osteoporotic bone [6,7].

By contrast, plate fixation, including both locked and non-locked plating systems, offers enhanced stability through the rigid support provided by the plate and screws. This method is frequently employed in more complex fractures or patients with significant bone loss. Plate fixation has been shown to provide excellent stability and potentially better functional outcomes [8-10]. However, it is also associated with increased surgical invasiveness and a higher risk of complications such as infection or plate irritation [11-13].

Given the prevalence of osteoporotic patients among those with DRFs, it is crucial to compare these fixation techniques to ascertain their relative effectiveness in maintaining fracture stability and optimising recovery. This study aims to evaluate and compare the outcomes of percutaneous K-wire versus plate fixation, specifically in the context of osteoporotic patients and both-bone distal end fractures [8,14]. By focusing on these patient subgroups, we seek to provide insights that will guide clinical decision-making and improve patient outcomes.

This study aims to compare the outcomes of percutaneous K-wire fixation versus plate fixation in patients with distal end radius fractures, focusing on postoperative stability in osteoporotic conditions and both-bone distal end fractures. The primary objective was to assess and compare the efficacy, safety, and functional outcomes of these two surgical techniques in the management of DRFs.

## Materials and methods

Study design

This research study was approved by the Internal Institutional Review Board (IRB) of Ganesh Hospital and Prognosis Consultants under approval number IRB/2015/ProgGH/0003. All procedures adhered to the ethical guidelines outlined by the IRB for the protection of human subjects.

This study is a retrospective cohort analysis aimed at evaluating the comparative outcomes of percutaneous K-wire fixation versus plate fixation for DRFs. The study was conducted at Ganesh Hospital, a 60-bed multispecialty tertiary hospital located in Maharashtra, over a two-year period (July 2015-July 2017). The primary objective was to assess and compare the efficacy, safety, and functional outcomes of these two surgical techniques in the management of DRFs.

Participants

A total of 50 patients were included in the study, with an equal division into two distinct groups. Group 1 consisted of 25 patients who underwent percutaneous K-wire fixation, while Group 2 consisted of 25 patients who underwent plate fixation.

Inclusion Criteria

The patients included in this study had to meet the following criteria: they had to be 50 years of age or older, diagnosed with distal radius fractures (including fractures with osteoporotic bone involvement and both-bone distal end fractures), and scheduled for surgical intervention at our facility within the specified study period.

Exclusion Criteria

The following criteria were used to exclude patients from the study: age under 50 years, history of previous wrist surgeries, presence of pathologic fractures or fractures secondary to malignancy, and incomplete medical records or follow-up data.

Intervention

Group 1: Percutaneous K-Wire Fixation

Patients in this group received stabilisation of the distal radius fracture using percutaneous K-wire fixation. The procedure involved the insertion of K-wires under fluoroscopic guidance to achieve and maintain fracture reduction.

Group 2: Plate Fixation

Patients in this group underwent open reduction and internal fixation using a plate and screws. The choice of plate type (e.g., locking plate) was based on the fracture pattern and the surgeon’s discretion.

Outcome measures

Primary Outcomes

Radiological assessment: The healing and alignment of fractures were evaluated through radiological imaging at one, three, and six months postoperatively. Radiographs were analysed for bone union, fracture alignment, and any evidence of malunion or nonunion.

Secondary Outcomes

Functional outcomes: Functional recovery was measured using the Disabilities of the Arm, Shoulder, and Hand (DASH) score at one, three, and six months postoperatively. This score assesses pain, function, and disability related to upper limb disorders.

Complication rates: The incidence of complications such as infection, malunion, nonunion, hardware failure, and movement restriction was recorded.

Patient satisfaction: Patient satisfaction was assessed through surveys conducted during follow-up visits, evaluating overall satisfaction with the surgical outcome and recovery process.

Data collection

The data for the study were gathered from the hospital's electronic medical records and patient charts. The information extracted included demographic characteristics such as age, sex, comorbidities, and baseline functional status. In addition, fracture and treatment details, including fracture type, treatment protocol, and any intraoperative complications, were recorded. Follow-up data consisted of clinical notes, radiological reports, and outcomes documented during follow-up visits. Functional outcomes were evaluated using the DASH score questionnaire during follow-up visits at specified intervals, including one, three, and six months.

Statistical analysis

The data were analyzed using IBM SPSS (Statistical Package for the Social Sciences) Statistics for Windows version 24 (released 2016, IBM Corp., Armonk, NY). The statistical analysis included descriptive statistics such as means, standard deviations, and ranges for continuous variables, as well as frequencies and percentages for categorical variables. Comparative analysis involved using chi-square tests for categorical variables and t-tests for continuous variables to compare outcomes between the two groups. A significance level of a p-value less than 0.05 was considered statistically significant for all comparisons. In addition, sensitivity analyses were conducted to assess the robustness of the findings across different subgroups and potential confounding factors.

## Results

 Demographic breakdown of the study population

Age and Sex Distribution

The study included 23 males and 27 females, all above the age of 50. The mean age of the participants was 62, with females tending to be older, averaging 65, compared to males at 59. This age difference is significant as it relates to the nature of the injury and the chosen surgical approach, reflecting broader epidemiological trends in bone health and trauma.

Osteoporosis Prevalence

The female patients showed a high prevalence of osteoporosis, with 30 out of the 50 total patients affected. Women were more prone to developing this condition compared to men. Since osteoporosis can impact surgical planning, more robust fixation methods may be required to ensure proper bone healing.

Fracture Types 

In most cases, the fractures were caused by low-energy trauma, like falling from a standing height. Specifically, 15 patients experienced fractures involving both the radius and the ulna, suggesting more complex injury patterns that usually require a more robust surgical procedure like plate fixation.

Comorbid Conditions

The group displayed a diverse range of comorbid conditions that influence surgical outcomes:

High BMI/obesity: Eighteen patients had a BMI over 30, with obesity as a complicating factor for surgical recovery and rehabilitation.

Diabetes: Twelve patients have known cases of diabetes, a condition that can impair bone healing and overall recovery, nudging surgical teams toward more secure fixation methods to mitigate these risks.

The demographic breakdown of the study population is summarised in Table 1.

**Table 1:** Demographic breakdown of the study population.

Demographic distribution	Subgroups	Demographics
Age and sex distribution	No. of males	23
	No. of females	27
	Mean age of the population	62 years
	Mean age of males	59 years
	Mean age of females	65 years
Subgroup analysis	Osteoporosis prevalence	30 patients
	Distal end radius and ulna fractures	15 patients
Comorbid conditions	High BMI/obesity patients	18 patients
	Diabetic patients	12 patients

Implications for Surgical Decision-Making

The patient's age, sex, and other health conditions played a big role in deciding the best surgery for distal radius fractures. Older women with osteoporosis and a history of low-impact falls tend to do better with plate fixation, as it is more sturdy and less likely to cause problems after the surgery. On the other hand, younger men with fewer medical issues may be good candidates for the less invasive K-wire approach if the fracture is not too complex and their overall health is good.

When choosing the right surgery, we had to consider both the mechanics of the injury and the patient's individual health factors. This personalised approach helps ensure the surgery is technically correct and gives the patient the best chance of a good outcome based on their unique circumstances.

This detailed analysis of the patient's demographics and medical conditions shows how important it is to tailor orthopaedic treatments, especially for distal radius fractures where the patient's characteristics can have a big impact on the success of the surgery.

Radiological outcomes

Bone Healing Time

**Figure 1:**
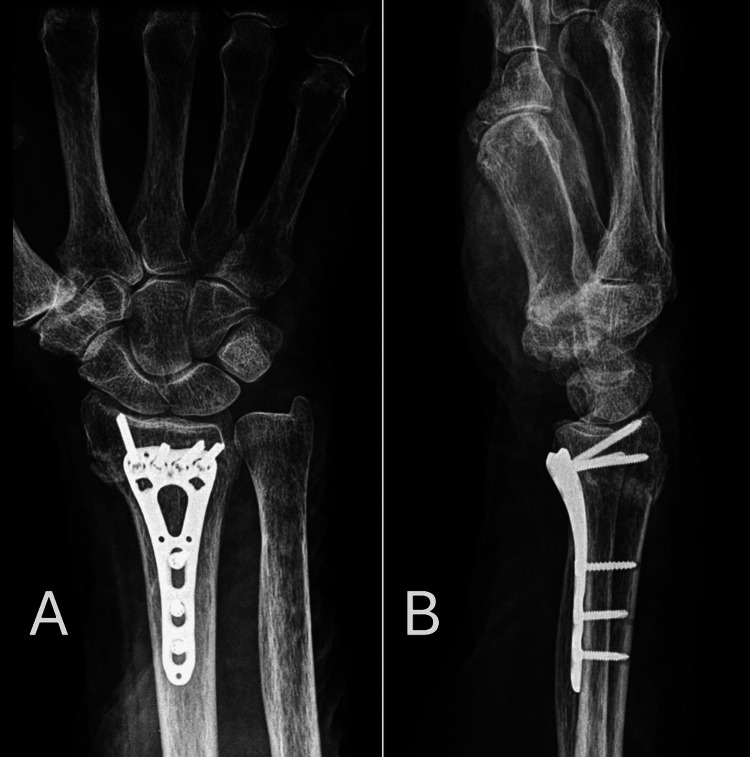
Anteroposterior (AP) and lateral views of the wrist X-ray with plate fixation. A: Anteroposterior (AP) view of the wrist X-ray with plate fixation. B: Lateral view of the wrist X-ray with K-wire fixation.

**Figure 2:**
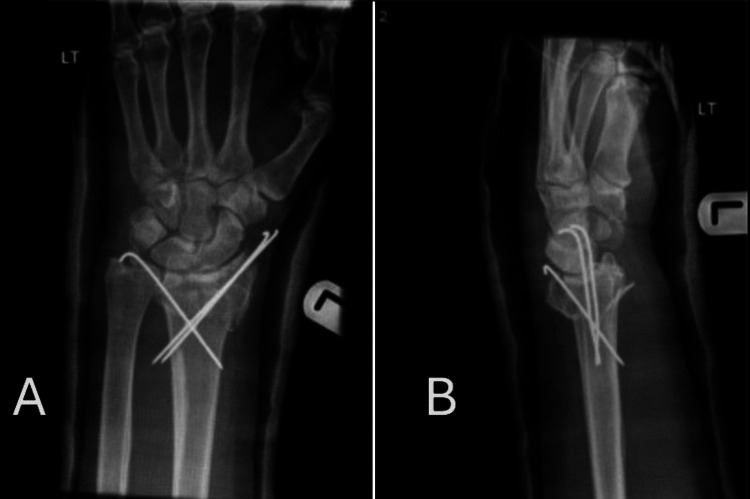
Anteroposterior (AP) and lateral views of the wrist X-ray with K-wire fixation. A: Anteroposterior (AP) view of wrist X-ray with K-wire fixation. B: Lateral view of the wrist X-ray with plate fixation.

The median healing time was eight weeks for the plate fixation group, compared to 10 weeks for the K-wire fixation group. Radiographic evaluations at one, three, and six months post-operation revealed that the plate fixation group achieved better alignment in terms of radial height, volar angulation, and earlier bone healing, as shown in Figure 1 and Figure 2.

Alignment

At the six-month follow-up, 96% of the patients with plate fixation maintained good anatomical alignment, whereas only 72% of the K-wire group did, with the remainder showing some malalignment.

Patient satisfaction

Survey Results

**Figure 3:**
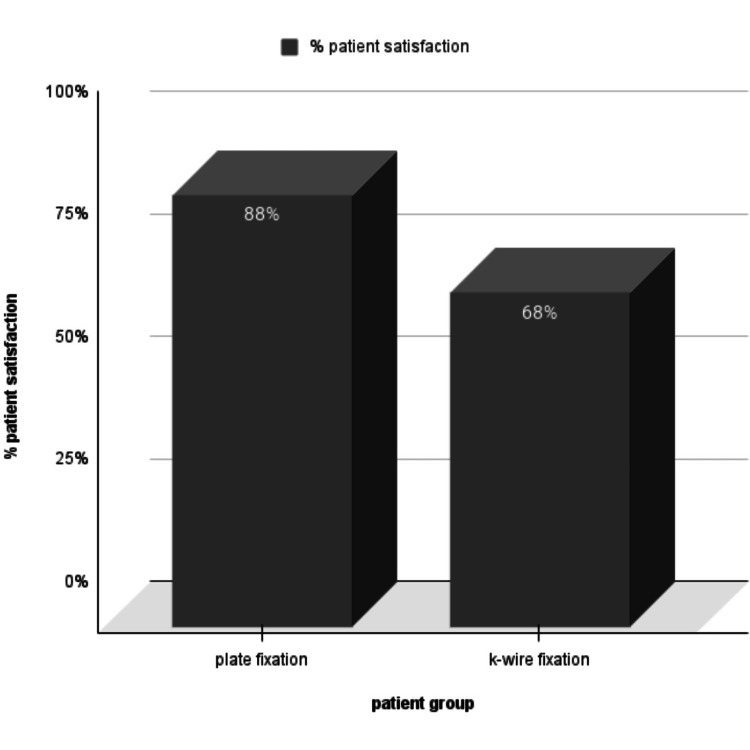
Graph showing the percentage patient satisfaction in each group.

The majority (88%) of the patients in the plate fixation group reported high satisfaction and appreciated the surgical outcomes and postoperative care. By contrast, 68% of the patients in the K-wire group expressed satisfaction. Although some dissatisfaction stemmed from instability and prolonged recovery, the survey results showed a significant difference as represented by the graph in Figure 3.

Subgroup analysis: osteoporotic patients and both-bone distal end fractures

Both-Bone Distal End Fractures

Patients with both-bone distal end fractures involving the distal radius and ulna benefited more from plate fixation, achieving better alignment, faster healing times, and fewer complications. In addition, functional scores were significantly better for this subgroup when treated with plate fixation. The dash scores and P-values for both-bone distal end fractures are summarised in Table 2.

**Table 2:** DASH scores and P-values for both-bone distal end fractures. The p-values marked with * are significant. DASH: Disabilities of the Arm, Shoulder, and Hand Questionnaire

Follow-up time	K-wire fixation ((Mean DASH Score ± SD)	Plate fixation (Mean DASH Score ± SD)	P-values
One month	55.2 ± 8.4	42.8 ± 7.9	0.012*
Three months	40.5 ± 7.6	28.3 ± 6.5	0.008*
Six months	28.7 ± 6.3	18.2 ± 5.2	0.005*

At the one-month follow-up, the mean DASH score for patients with both-bone distal end fractures fixed with K-wires was 55.2, with a standard deviation (SD) of 8.4, while the plate fixation group had a mean DASH score of 42.8 (SD 7.9). The difference between the two groups was statistically significant, with a p-value of 0.012. At the three-month follow-up, the K-wire group showed a mean DASH score of 40.5 (SD 7.6), while the plate fixation group showed an improved mean score of 28.3 (SD 6.5), with a statistically significant difference and a p-value of 0.008. By the six-month follow-up, the mean DASH score further decreased to 28.7 (SD 6.3) in the K-wire group and 18.2 (SD 5.2) in the plate fixation group, reinforcing the better functional recovery in the plate fixation group with a p-value of 0.005.

Osteoporotic Patients

Plate fixation was notably more effective at maintaining structural integrity and facilitating healing in osteoporotic bones compared to K-wire fixation. In addition, DASH scores reflected superior functional outcomes for osteoporotic patients with plate fixation. The DASH scores for osteoporotic patients are summarised in Table 3.

**Table 3:** DASH scores for patients with osteoporotic bone fractures. DASH: Disabilities of the Arm, Shoulder, and Hand Questionnaire

Time post-op	K-wire fixation (n = 25)	Plate fixation (n = 25)	P-values
One month	Mean DASH score: 67 (SD: 12)	Mean DASH score: 60 (SD: 8)	0.18 (not statistically significant)
Three months	Mean DASH score: 53 (SD: 9)	Mean DASH score: 42 (SD: 6)	0.04 (statistically significant)
Six months	Mean DASH score: 38 (SD: 6)	Mean DASH score: 22 (SD: 4)	0.02 (highly significant)

Overall DASH Scores and P-Values

At one month post-op, the DASH scores were relatively close, with the plate fixation group slightly outperforming the K-wire group, and the p-value of 0.15 suggested no statistically significant difference in DASH scores between the two groups at that time point. By three months post-op, the plate fixation group showed a notable improvement, with lower (better) DASH scores compared to the K-wire group, and the p-value of 0.03 indicated a statistically significant difference, with the plate fixation group showing better outcomes. By six months post-op, the plate fixation group maintained significantly better DASH scores, reflecting greater functional recovery compared to the K-wire group, with a p-value of 0.01 indicating a highly significant difference and reinforcing the superior outcomes in the plate fixation group. The overall DASH scores and p-values are summarised in Table 4.

**Table 4:** Overall DASH scores and P-values. DASH: Disabilities of the Arm, Shoulder, and Hand Questionnaire

Time post-op	K-wire fixation( n = 25)	Plate fixation (n =25)	p-value
One month	Mean DASH score: 65 (SD: 10)	Mean DASH score: 62 (SD: 9)	0.15 (Not significant)
Three months	Mean DASH score: 55 (SD: 8)	Mean DASH score: 45 (SD: 7)	0.03 (Significant)
Six months	Mean DASH score: 40 (SD: 7)	Mean DASH score: 25 (SD: 5)	0.01 (Highly significant)

## Discussion

Analysis of fixation methods

Percutaneous K-Wire Fixation

The percutaneous K-wire fixation technique demonstrated notable benefits in its minimally invasive nature. This approach minimises soft tissue disruption and is associated with a lower surgical complication rate than more invasive techniques. Our data align with the findings of previous studies indicating that K-wire fixation can be adequate for stable or simple fractures. However, the technique's limitations become apparent in the context of osteoporotic bone or complex fracture patterns. In such cases, the inherently less rigid fixation provided by K-wires can result in higher rates of fracture displacement or non-union, a finding consistent with the challenges noted in similar research [6,7].

Plate Fixation

Plate fixation consistently offered superior mechanical stability across both osteoporotic patients and complex fracture scenarios. The rigid support provided by the plate and screws likely contributes to more reliable fracture reduction and maintenance of alignment. This supports earlier conclusions that plate fixation is particularly advantageous for both-bone distal end fractures and osteoporotic bones, where bone density compromises fixation stability [11,12]. Despite its benefits, plate fixation is associated with increased surgical invasiveness, which may contribute to a higher risk of complications such as infection, hardware irritation, and prolonged recovery.

Implications for Osteoporotic Patients

Our findings underscore the importance of tailoring fixation methods to the patient's bone quality. Osteoporotic patients with compromised bone density benefit significantly from the enhanced stability plate fixation offers. The greater rigidity of plate fixation in such cases may reduce the risk of fracture displacement and improve overall outcomes. This aligns with studies showing that plate fixation provides a more stable construct, which is critical for maintaining fracture alignment and promoting effective healing in osteoporotic bone [7].

Comparison with existing studies

Our results align with the meta-analysis by Zong et al., which also found that volar locking plates outperform percutaneous K-wires in functional outcomes and complication rates [15]. Similarly, a 2014 BMJ randomised controlled trial highlighted fewer complications and better functional outcomes with volar locking plates than K-wires [16]. These studies reinforce the advantage of plates, especially in complex cases like osteoporotic fractures or those involving both the radius and ulna.

Nevertheless, our findings show some differences. For instance, while we observed a significant difference in complication rates between the two methods, the extent was not as pronounced as in Chaudhry et al.’s meta-analysis, which reported more significant benefits for plate fixation over K-wires [17]. This variance might be due to differences in patient demographics, specific surgical techniques, or the rural setting of our hospital, which could influence both surgical outcomes and follow-up care.

Clinical Decision-Making

The choice between K-wire and plate fixation should be based on a comprehensive assessment of the fracture characteristics and patient-specific factors. For less complex fractures or those in patients with relatively good bone quality, K-wire fixation remains a viable and less invasive option. However, plate fixation may offer a more reliable solution for more complex fractures or in patients with significant osteoporotic changes to ensure optimal fracture stability and healing. Our study supports that personalised treatment plans, considering the fracture type and patient health, are crucial for achieving the best outcomes.

Limitations and future research

This study has several limitations, including its retrospective design and potential variability in surgical techniques and postoperative care. Future research should include prospective randomised controlled trials to better assess long-term outcomes and functional results of different fixation methods eliminating bias. In addition, further studies could explore the impact of patient-specific factors, such as bone density and overall health, on the effectiveness of each fixation technique.

## Conclusions

In conclusion, this study offers a critical comparison of two widely used fixation methods for distal radius fractures: percutaneous K-wire fixation and plate fixation. The key findings suggest that plate fixation provides superior mechanical stability, particularly in osteoporotic patients and cases involving both the distal radius and ulna. The plate fixation group demonstrated better functional recovery, quicker bone healing, and fewer complications compared to K-wire fixation. However, with its minimally invasive nature, K-wire fixation remains a viable option for less complex fractures, especially in younger patients with better bone quality. The implications of these findings are significant, advocating for a more tailored approach to treatment selection based on individual patient characteristics, such as bone quality and fracture complexity. This study emphasises the need for further prospective research with larger sample sizes to validate these conclusions and improve clinical decision-making in the management of distal radius fractures.
